# Pro-renin receptor suppresses mitochondrial biogenesis and function via AMPK/SIRT-1/ PGC-1α pathway in diabetic kidney

**DOI:** 10.1371/journal.pone.0225728

**Published:** 2019-12-04

**Authors:** Safia Akhtar, Helmy M. Siragy

**Affiliations:** Department of Medicine, University of Virginia, Charlottesville, Virginia, United States of America; University of Louisville, UNITED STATES

## Abstract

Abnormal mitochondrial biogenesis and function has been linked to multiple diseases including diabetes. Recently, we demonstrated the role of renal (Pro)renin receptor (PRR) in the dysregulation of mitochondria. We hypothesized that PRR contributes to the reduction of mitochondrial biogenesis and function in diabetic kidney via PGC-1α/AMPK/SIRT-1 signaling pathway. *In vivo* and *in vitro* studies were conducted in C57BL/6 mouse and mouse renal mesangial cells (mRMCs). Control and streptozotocin-induced diabetic mice were injected with scramble or PRR shRNA and followed for a period of eight weeks. PRR mRNA and protein expression increased by 44% and 39% respectively (P<0.05) in kidneys of diabetic mice, and in mRMCs exposed to high glucose by 43 and 61% respectively compared to their respective controls. These results were accompanied by reduced mRNA and protein expressions of PGC-1α (67% and 75%), nuclear respiratory factors (NRF-1, 48% and 53%), mitochondrial transcriptional factor A (mtTFA, 56% and 40%), mitochondrial DNA copy number by 75% (all, P<0.05), and ATP production by 54%, respectively in diabetic kidneys and in mRMCs exposed to high glucose. Compared to non-diabetic control mice, PRR knockdown in diabetic mice and in mRMCs, not only attenuated the PRR mRNA and protein expression but also normalized mRNA and protein expressions of PGC-1α, NRF-1, mtTFA, mitochondrial DNA copy number, and ATP production. Treatment with AMPK inhibitor, Compound C, or SIRT-1 inhibitor, EX-527, alone, or combined with PRR siRNA caused marked reduction of mRNA expression of PGC-1α, NRF-1 and mtTFA, and ATP production in mRMCs exposed to high glucose. In conclusion, our study demonstrated the contribution of the PRR to the reduction of mitochondrial biogenesis and function in diabetic kidney disease via decreasing AMPK/SIRT-1/ PGC-1α signaling pathway.

## Introduction

Diabetic kidney disease (DKD) is one of the major complications of diabetes and leads to end-stage renal disease [1, 2]. The common pathological features of DKD are mesangial cell proliferation, glomerular hypertrophy, and thickening of the glomerular basement membrane [3]. These structural changes, however, are preceded by early metabolic changes, such as deficient oxygen handling, mitochondrial dysfunction and increased oxidative stress [4, 5]. Diabetes is also associated with high mutation rate of mitochondrial DNA (mtDNA) [6–9] leading to lower mitochondrial content [6, 10]. The kidney has the greatest density of mitochondria per tissue mass. Thus, impaired mitochondria could play a critical role in the pathogenesis of DKD. However, the precise mechanism(s) involving the influence of diabetes on mitochondria biogenesis and the development of DKD remains poorly understood.

(Pro)renin receptor (PRR) is highly expressed in different kidney components including mesangial cells, podocyte, endothelial cells, proximal tubular cells, and collecting duct cells [11–13]. PRR is upregulated in kidneys of diabetic mice and renal mesangial cells exposed to high glucose concentration and contributes to the development of DKD. The development of renal inflammation and albuminuria were notably ameliorated with PRR knockdown in diabetic mice [12, 14]. Recently, studies from our laboratory revealed diabetes-induced overexpression of PPR in the renal mitochondria and associated with increased mitochondrial oxidative stress and dysfunction. Collectively, these striking findings reinforce the potential role of PRR in the development of renal mitochondrial dysfunction. A loss of mitochondrial biogenesis or function could be the basis or consequence of reduced organ function. Therefore, mitochondrial protection could become a new approach to the treatment of DKD. However, how mitochondrial biogenesis is impaired and by which mechanism it is being regulated in the DKD remains largely unknown. The aim of the current study is to evaluate whether PRR modulates mitochondrial function and biogenesis in the DKD and elucidate the involved mechanisms.

Mitochondrial biogenesis is dependent on different signaling cascades and transcriptional complexes. It is tightly controlled by a master regulator PGC-1α, nuclear respiratory factors (NRF-1 and NRF-2) and mitochondrial transcriptional factor A (mtTFA), which in turn activate expression of nuclear and mitochondrial genes encoding mitochondrial proteins. AMP-activated protein kinase (AMPK) is a serine/threonine protein kinase and silent information regulator-1 (SIRT-1) have emerged as master sensors of cellular energy balance. In addition, growing body of evidence suggest that AMPK and SIRT-1 promote mitochondrial biogenesis and oxidative capacity via regulating PGC-1α and prevent the mitochondrial dysfunction [15, 16]. AMPK appears to be the predominant regulator of mitochondrial biogenesis and is essential for the beneficial metabolic effects and glucose homeostasis [17].

Thus, the aim of the present study is to investigate the role of PRR in regulating the activity of PGC-1α, and thus, the mitochondrial biogenesis and mitochondrial function in the DKD. In addition, we further propose to elucidate the molecular signals that cause impairment of mitochondrial biogenesis. In the current study, we hypothesized that PRR contributes to the development of DKD by downregulating mitochondrial biogenesis and function via AMPK/SIRT-1/ PGC-1α signaling pathway.

## Materials and methods

### Animals

The University of Virginia Animal Care and Use Committee (ACUC) approved all of the experimental protocols. In brief, eight week old male C57BL/6 (BL6) mice purchased from Jackson Laboratory were maintained at 23°C and a 12:12-h light-dark cycle with free access to water and a standard chow diet. PRR shRNA or scrambled shRNA (Viral vector core, University of Iowa, Iowa City, IA) were directly microinjected into the right kidney of the mice. Animals were divided randomly into four groups: normoglycemic control group injected with either scrambled shRNA (Veh + Scr shRNA, n = 6) or PRR shRNA (Veh + PRR shRNA, n = 6), and streptozotocin (STZ) induced diabetes mellitus (DM) groups injected with either scrambled shRNA (STZ + Scr shRNA, n = 6) or PRR shRNA (STZ + PRR shRNA, n = 6). Diabetic mice received 55 mg/kg STZ (STZ; Sigma-Aldrich, Saint Louis, MO), intraperitoneally, for 5 consecutive days, and nondiabetic control mice received an equal volume of vehicle (0.9% NaCl). Body weight was measured at baseline and at the end of the study. Blood glucose level was measured at weeks 1, 5, and 8 from tail vein using a glucometer. For urine collections, mice were placed in individual metabolic cages for a period of 24h in the last week of study and urine samples were kept at −80°C until assayed. Urinary albumin was determined by using a commercial mice albumin ELISA kit (Exocell, Philadelphia, PA), and urine creatinine was assessed by creatinine assay kit (Cayman Chemical, Ann Arbor, MI) to calculate urine albumin-creatinine ratio (UACR). At the end of the study, all mice were sacrificed and the remaining right kidney was harvested for protein and RNA extraction, immunostaining as well as morphological examinations.

### PRR short hairpin RNA (shRNA) transfection in the animals

Animal surgery was performed under sterile condition on all animals under ketamine/xylazine (80/10 mg/kg, IP) anesthesia to minimize pain and distress. The efficacy of anesthesia was determined by the absence of pain reflex elicited by pinching the tail, as well as by the absence of palpebral reflex by touching the eyelid. Eye lubricant ointment was applied to both eyes of the mouse to prevent eye dryness while under anesthesia. Mouse was placed on a warming pad (set at 37°C) to maintain the body temperature around 37°C during the procedure. A right kidney was exposed and a 50 μl PRR shRNA or scrambled shRNA (Viral vector core, University of Iowa, Iowa City, IA) were directly microinjected into the right kidney of the mice using a minipump at 3 μl/min. At the end of the procedure, muscle incision was closed with 4-0 silk suture string and then close the skin incision with 3-0 silk suture string. Animals were housed separately in a cage with a heat lamp over the cage so that the bedding area is close to 37°C before placing the animals in individual cages. All animals were monitered until they have regained sufficient consciousness. Easy access to water and food were allowed during recovery. All mice received postoperative analgesia every 12 h for the first 3 days via subcutaneous injection of 0.1 mg/kg buprenorphine to prevent infection and pain. After surgery, animals were monitered twice daily for three days, and then once a day for the duration of the experiment. Animals drinking, eating, walking patterns, awkward gait, hunched back, and aggressive behavior were monitored during recovery period. Mouse were immediately euthanize if it shows any sign of seizure, coma, untreatable infection, difficulty to walk, loss of gait, unable to eat and drink. At the end of the experiment, all animals were sacrificed with ketamine/xylazine (80/10 mg/kg, IP) followed with cervical dislocation under anesthesia.

### Cell culture

Mouse renal mesangial cells (mRMCs) were obtained from the American Type Culture Collection (ATCC, Manassas VA) and cultured according to ATCC recommended protocol. The cells were cultured in medium containing 25 mM D-glucose (high glucose) for experiments groups and 5 mM D-glucose plus 20 mM L-glucose (normal glucose) for control groups for 3 days in a 6 well plate.

### PRR siRNA transfection to the cells

After 3 days exposure to high and normal glucose, cells were transfected with either Scr siRNA or PRR siRNA for 6 h in transfection reagent. After incubation, the media was changed to NG or HG medium for overnight. At the end of the experiments, cells were harvested for total RNA and protein extraction. Transfection of PRR siRNA or scrambled siRNA was performed using the siLentFect lipid reagent (Bio-Rad, Hercules, CA) according to the manufacturer's instructions. Ten nmol of PRR siRNA - SMARTpool (Accell mouse, Thermo Scientific Dharmacon Research Inc, USA, target sequences: 5′-CGAAUAGAUUGAAUUUUCC-3′; 5′-CGGUAUACCUUAAGUUUAU-3′; 5′-UGGUUUAGUAGAGAUAUUA-3′; 5′-GGACCAUCCUUGAGGCAAA-3′) was used for each well. A scrambled siRNA (QIAGEN, Valencia, CA, target sequences: 5′-AATTCTCCGAACGTGTCACGT-3′), which was confirmed as non-silencing double-stranded RNA, was used as control for siRNA experiments. Our previous studies indicated that 10 nM siRNA duplex resulted in a maximal suppression of PRR mRNA for 48 hrs and of PRR protein expression for 72hrs [14]. For treatment experiments, cells were starved for 6 h and then AMPK inhibitor (Compound C; 5μM, Abcam), in serum-free medium, was added to the cells for 6 hours, prior to cell harvesting. Whereas, a selective SIRT-1 inhibitor (EX-527; 100 nM, Santa Cruz Biotechnology, Santa Cruz, CA) was added to serum-free medium 30 min before the end of serum starvation. At the end of the treatment, cells were harvested for protein and RNA extraction. For combined treatment, AMPK and SIRT-1 was applied to PRR siRNA treated cells respectively, for 6 h and 30 min, prior to cell harvesting.

### Protein extraction and western blot analysis

Protein levels were determined using western immunoblotting on whole cell lysates from both *in vivo* and *in vitro*. For protein extraction, whole kidney homogenates or cells were lysed in the presence of protease inhibitor cocktail (Thermo Scientific). Clear protein extracts were obtained by centrifugation at 12,000 g for 10 min at 4°C. Protein concentrations were determined by BCA protein assay, and 20–40 μg of protein mixed with loading buffer was loaded per lane. Western blot analysis was performed as described previously [14]. In brief, 20 μg of total proteins were subjected to SDS-PAGE, transferred onto polyvinylidene difluoride (PVDF) membrane filters (Bio Rad). PVDF membranes were blocked with 5% dry milk for 1 h. Membranes were incubated in the primary antibody overnight at 4°C. The following antibodies were used in Western blotting: PRR [18] (1:1,000; anti-ATP6IP2/ab40790, Abcam, MA), PGC-1α [19] (1:1000, ab54481, Abcam), NRF-1 [20] (1:1000, ab34682, Abcam, MA), mtTFA [21] (1:1000, ab131607, Abcam), p-AMPK [22, 23] (1:1000, 2535S, CST), t-AMPK [23] (1:1000, 2532S, CST), SIRT-1 [24] (1:1000, 9475S, CST). The membranes were then incubated with the corresponding secondary antibody (1:2000, horseradish peroxidase-conjugated anti-rabbit) in TBST-5% nonfat milk for 1 h at room temperature, and the immunoreactive bands were visualized followed by incubation with horseradish peroxidase-labeled IgG (1:5000). The immunoreactive bands were detected by chemiluminescence methods and visualized on ChemiDoc Imaging system (Life Science Research, Bio Rad, CA, USA). Densitometric analysis of the images was performed using the Image J software (NIH, Bethesda, MD, USA).

### Real-time PCR: Determination of mRNA expression

Total RNA was extracted using an Direct-zol RNA MiniPrep Kit (Zymo Research, Genesee Scientific, San Diego, CA, USA) according to the manufacturer's protocol. RNA concentration was measured by NanoDrop 1000 (Thermo Fisher Scientific, Waltham, MA, USA). Aliquots of total RNA (1 **μ**g) from each sample were utilized and single-stranded cDNA was synthesized using iScript cDNA Synthesis Kit (Bio-Rad Hercules CA, USA). PCR was performed with iQTM SYBR green supermix (Bio-Rad Hercules CA, USA), as the fluorescence indicator, according to the manufacturer's instructions. Expression levels of PRR, PGC-1α, NRF-1 and mtTFA mRNA were measured by a real-time RT-PCR iCycler according to the manufacturer's instructions (Bio-Rad, Hercules CA, USA). Primers sequences used in this study are as follows: PRR, forward 5’TTTGGATGAACTTGGGAAGC 3’, reverse 5’-CACAAGGGATGTGTCGAATG-3’; PGC-1α, forward 5’-AAACTTGCTAGCGGTCCTCA-3’, reverse 5’-TGGCTGGTGCCAGTAAGAG-3’, NRF-1 forward 5’-GCACCTTTGGAGAATGTGGT-3’ reverse 5’-GGGTCATTTTGTCCACAGAGA-3’, mtTFA forward 5’-CCTTCGATTTTCCACAGAACA-3’ reverse 5’-GCTCACAGCTTCTTTGTATGCTT-3’. Reactions were performed in duplicate, and threshold cycle numbers were averaged. The mRNA levels of target genes were normalized to the GAPDH mRNA levels.

### Quantitative real-time PCR for mitochondrial DNA content

Mitochondria DNA copy number was quantified by real time quantitative RT-PCR using the mtDNA Copy Number Kit (MCN3) (Detroit R&D, MI). DNA was extracted from frozen kidney tissues and from cultured mRMCs using the DNA isolation kit (Qiagen DNeasy blood and tissue kit). The total DNA concentration was determined using a NanoDrop 1000 (Thermo Fisher Scientific). MtDNA levels were quantitated by normalizing the mitochondrial gene (cytochrome b) to the nuclear gene (GAPDH). Evaluation of mtDNA content in tissue homogenate and cultured cells was performed by quantitative real-time PCR as described and expressed as mtDNA/nuclear DNA ratio.

### Immunohistochemical staining

Immunohistochemical staining was performed to determine the renal expression of PRR in the kidney tissue. 4-μm-thick sections were used. Heat-induced antigen retrieval was conducted in 10mM sodium citrate (pH 6.0). Endogenous peroxide activity was suppressed by 3% peroxide-methanol solution. VECTASTAIN® ABC KIT (Vector Laboratories, Burlingame, CA) was used for blocking and color reaction. Immunostaining was performed by incubating overnight at 4°C with primary antibody (PRR 1:100, Sigma Aldrich HPA003156) followed by 30min of incubation with a secondary antibody (1:1000, horseradish peroxidase-conjugated anti-rabbit) conjugated with biotin at room temperature.

For kidney tissue histology evaluation, **s**ections (4-μm thick) cut from 4% formalin-fixed, paraffin-embedded kidney samples were used for periodic acid-Schiff (PAS) staining.

### Assessment of ATP production

Renal mitochondrial ATP levels were measured by using a commercially available ATP assay kit (K354-100, Biovision, CA) according to manufacturer instructions. Kidney tissue samples were homogenized in ATP assay buffer and added into 96 well plate with the flat bottom. Enough reaction mix was added into the 96 well plate. After 30 min incubation at room temperature, absorbance was measured at optical density 570 nm in a microplate reader (Epoch, BioTek, Japan). ATP concentrations were calculated by the construction of a standard ATP calibration curve.

### Statistical analysis

Comparisons among different treatment groups were assessed by Student's *t-*test (2-tailed) or by one-way ANOVA when appropriate followed by a Tukey *post-hoc* test. Data are expressed as means ± SEM. P*<*0.05 is considered statistically significant.

## Results

### Blood glucose, body weight, 24-hour urinary albumin excretion, urinary albumin to creatinine ratio (UACR) and renal histology

**Fig 1:**
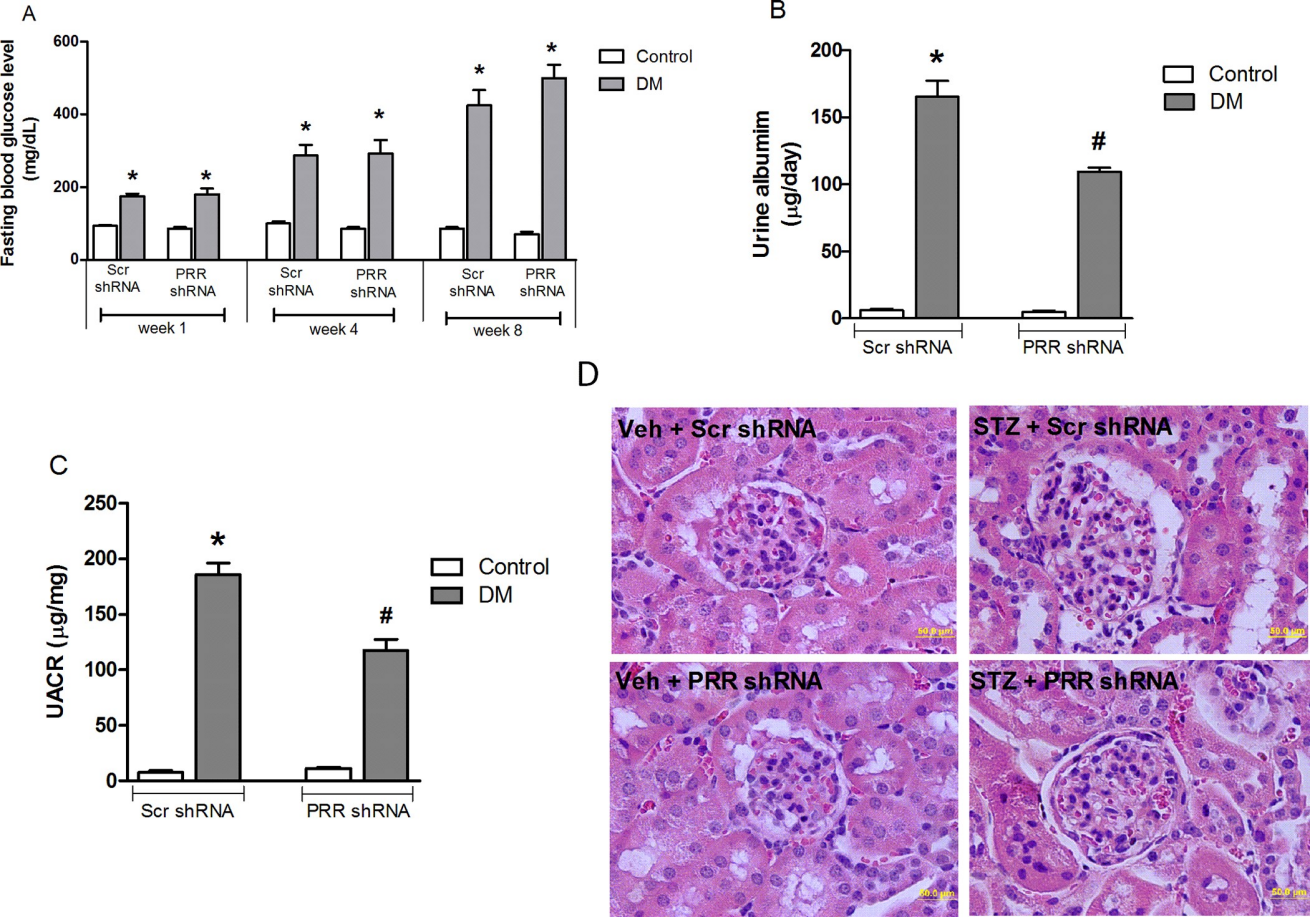
Fasting blood glucose levels in mice at weeks 1, 4 and 8 after diabetes induction. (A) 24-hour urinary albumin levels in mice (B) and urinary albumin to creatinine ratio (UACR) (C) in mice at the end of study (n = 8, each group). Representative images showing glomerular PAS staining in control and STZ-induced diabetic mice (D) treated with either scramble (Scr) shRNA or PRR shRNA respectively. Data presented as mean ± SEM, *P < 0.05 vs. Veh + Scr shRNA; #P < 0.05 vs. DM + Scr shRNA.

Compared to non-diabetic mice, STZ-induced diabetic mice had significantly (P<0.05; Fig 1A) higher fasting blood glucose levels. The 24-hour urinary albumin excretion (Fig 1B) and UACR ratio (Fig 1C), were markedly increased (P<0.05) in STZ-induced diabetic mice compared to non-diabetic mice. Compared to scramble shRNA treatment in diabetic mice, PRR shRNA treated diabetic mice had lower levels of 24-hour urinary albumin excretion and UACR ratio by 39 and 45% respectively (P<0.05). In addition, PAS staining (Fig 1D) showed normal glomerular structure in scramble shRNA control mice, whereas scramble shRNA treated diabetic mice exhibited glomerular hypertrophy and mesangial matrix expansion. PRR shRNA treatment reduced these histologic changes in diabetic mice.

### Expression of PRR in diabetic kidneys

**Fig 2:**
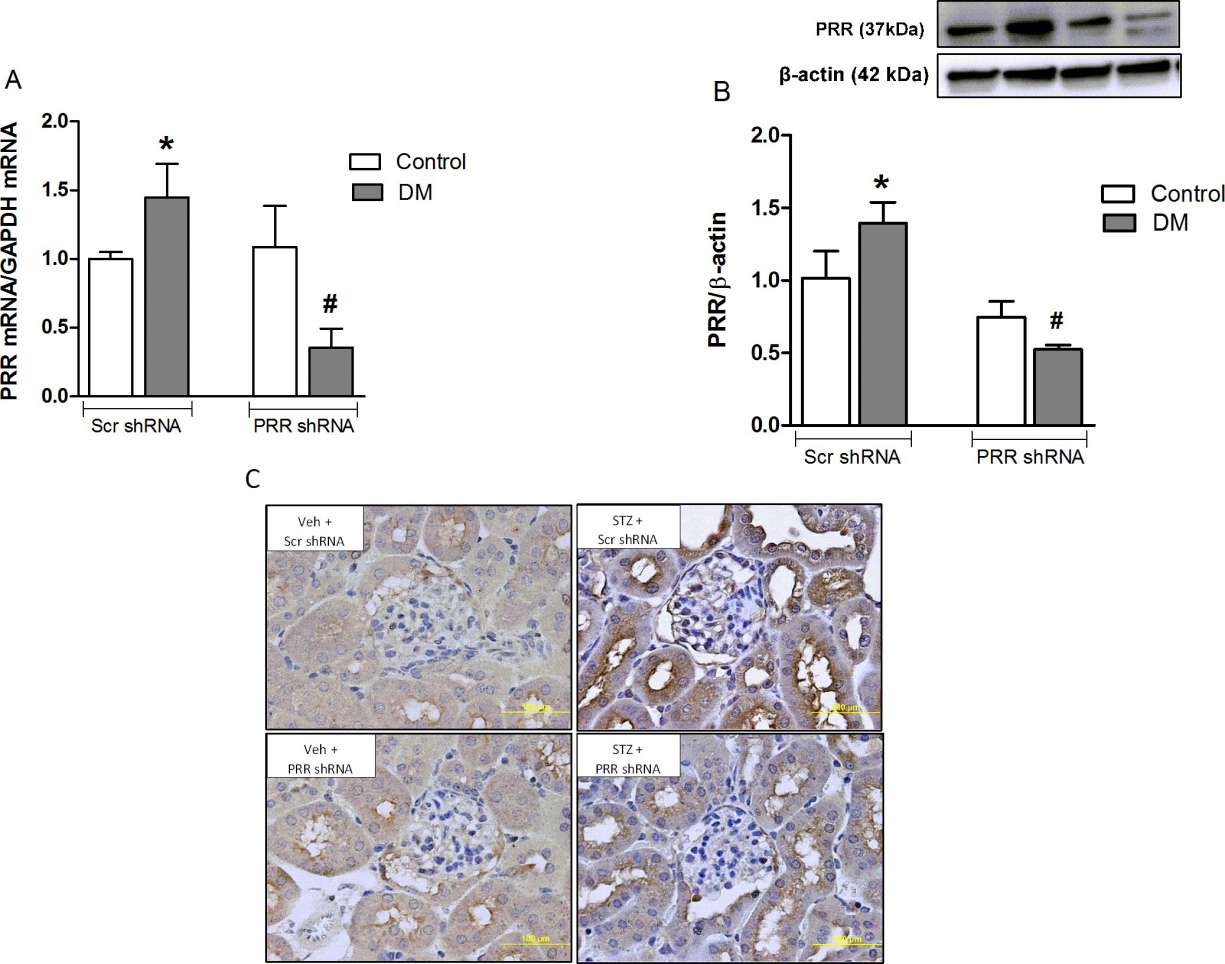
Renal expressions of (pro)renin receptor (PRR). (A) mRNA and PRR protein (B) in non-diabetic control mice (n = 6 each group), and streptozotocin (STZ)-induced diabetic mice (DM; n = 6) treated with PRR shRNA (DM + PRR + shRNA; n = 6 each group), after eight weeks of STZ-induction of diabetes. Representative images of PRR immunostaining (C) in renal cortex. Top row represents Vehicle + scramble (Scr) shRNA and lower row represents PRR shRNA treatment in non-diabetic and STZ induced diabetic mice respectively. Data are mean ± SEM. *P < 0.05 vs. Veh + Scr shRNA; #P < 0.05 vs. DM + Scr shRNA.

In non-diabetic mice, there were no changes in PRR mRNA and protein expressions in response to treatment with scramble or PRR shRNA. Compared to non-diabetic mice, STZ induced diabetic mice showed significant increase of the kidney PRR mRNA and protein by 44% and 39% respectively (P<0.05), whereas knockdown of PRR in diabetic animals significantly decreased PRR mRNA and protein expression of PRR by 75% and 62% (P<0.05), respectively compared to scramble shRNA-treated diabetic group (Fig 2A and 2B). Similarly, immunohistochemical staining also showed increased PRR staining in the renal cortex of scramble shRNA diabetic mice which was markedly decreased in PRR shRNA treated diabetic mice (Fig 2C).

### Effect of PRR on mitochondrial biogenesis marker PGC-1α, NRF-1 and mtTFA expression in diabetic kidneys

**Fig 3:**
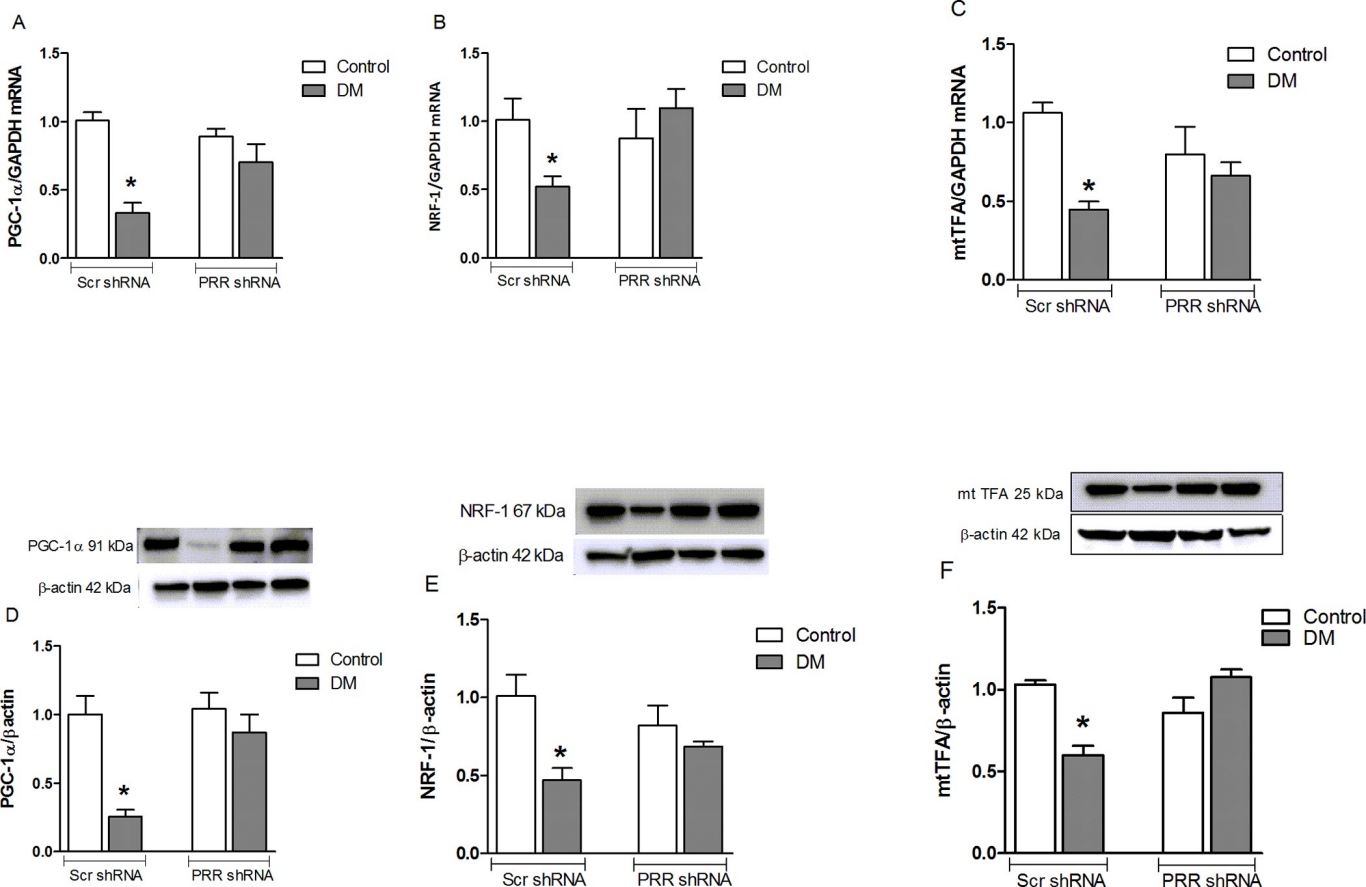
Renal expressions of PGC-1α, NRF-1 and mtTFA. (A-C) mRNA and (D-F) protein expressions in normoglycemic control mice treated with scramble (Scr) shRNA (n = 6 each group), and streptozotocin (STZ)-induced diabetic mice treated with PRR shRNA (DM + PRR shRNA; n = 6 each group), after eight weeks of STZ-induction of diabetes. Data are mean ± SEM. *P < 0.05 vs. Veh + Scr shRNA.

PGC-1α is a master transcription factor for the formation of more mitochondria by transactivating NRF-1, which lead to the increase in the expression of the gene encoding mtTFA, a key activator of mitochondrial gene transcription. We, therefore, measured these markers to determine whether mitochondrial biogenesis is suppressed in the diabetic kidney, and is dependent on PRR. Compared to non-diabetic mice kidney, there were significant decreases in mRNA expression of PGC-1α, NRF-1, and mtTFA by 67, 48 and 56% (Fig 3A–3C). respectively (P<0.05) and their protein expressions by 75, 53 and 40% (P<0.05), respectively in STZ-induced diabetic mice kidneys (Fig 3D–3F). Both mRNA and protein levels of PGC-1α, NRF-1 and mtTFA were normalized when diabetic mice were treated with PRR shRNA, confirming the role of PRR in the regulation of mitochondrial biogenesis.

### Effect of PRR on AMPK phosphorylation and SIRT-1 protein expression in the kidneys of diabetic mice and in mRMCs

**Fig 4:**
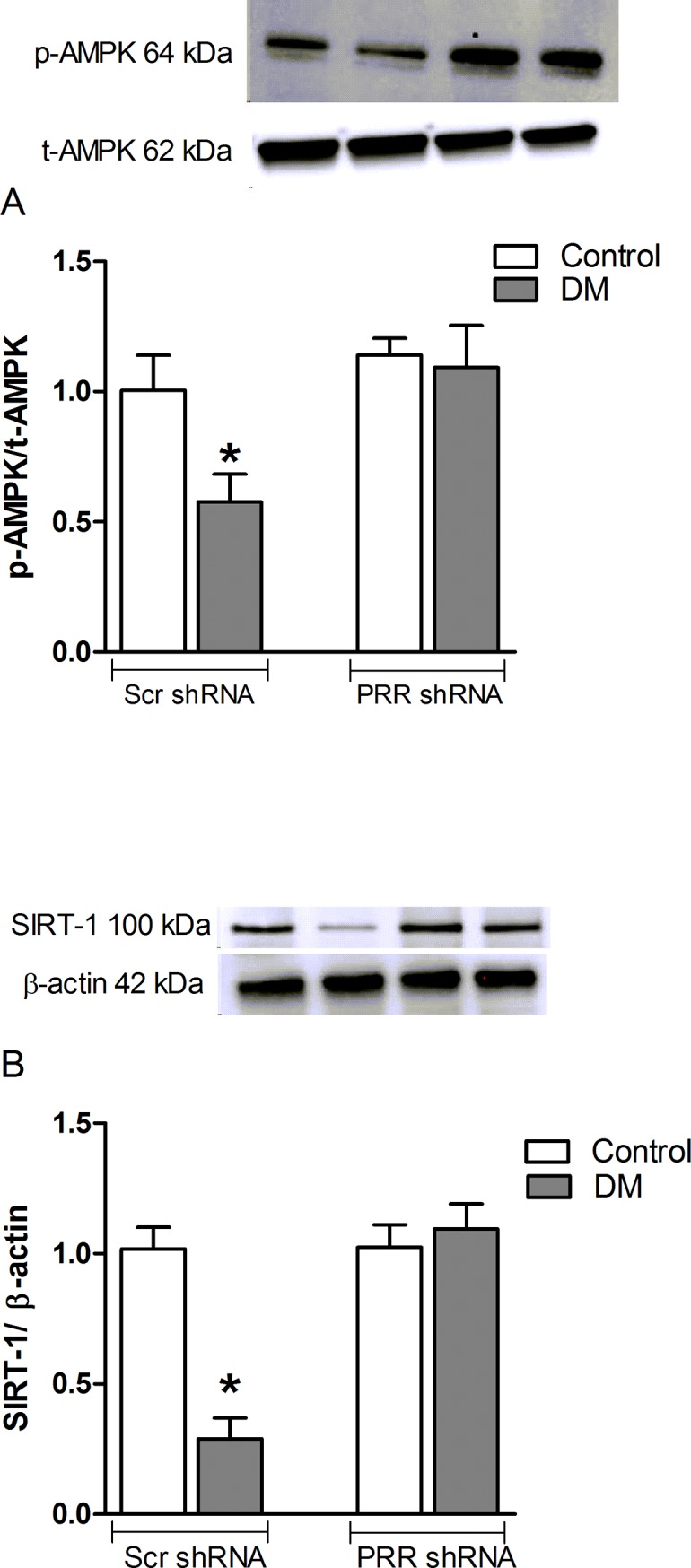
Western blot analysis of AMPK and SIRT-1 protein expression. pAMPK and total AMPK protein expression. (A) pAMPK and total AMPK and (B) SIRT-1 protein expression in renal cortex of normoglycemic control mice treated with scramble (Scr) shRNA (n = 6 each group), and streptozotocin (STZ)-induced diabetic mice (DM; n = 6 each group) treated with PRR shRNA (DM + PRR + shRNA; n = 6 each group), after eight weeks of STZ-induction of diabetes. Data are mean ± SEM. *P < 0.05 vs. Veh + Scr shRNA.

Compared to non-diabetic mice, STZ-induced diabetic mice showed noticeable decreases in pAMPK/tAMPK and SIRT-1 protein expression (Fig 4A and 4B) by 43 and 71% respectively (both, P<0.05). PRR shRNA reversed the diabetes-induced decrease of pAMPK/tAMPK and SIRT-1 protein expression (Fig 4A and 4B), and the levels were similar to that of PRR shRNA treated non-diabetic animals.

### Mitochondrial DNA copy number and ATP content in the diabetic kidneys

**Fig 5:**
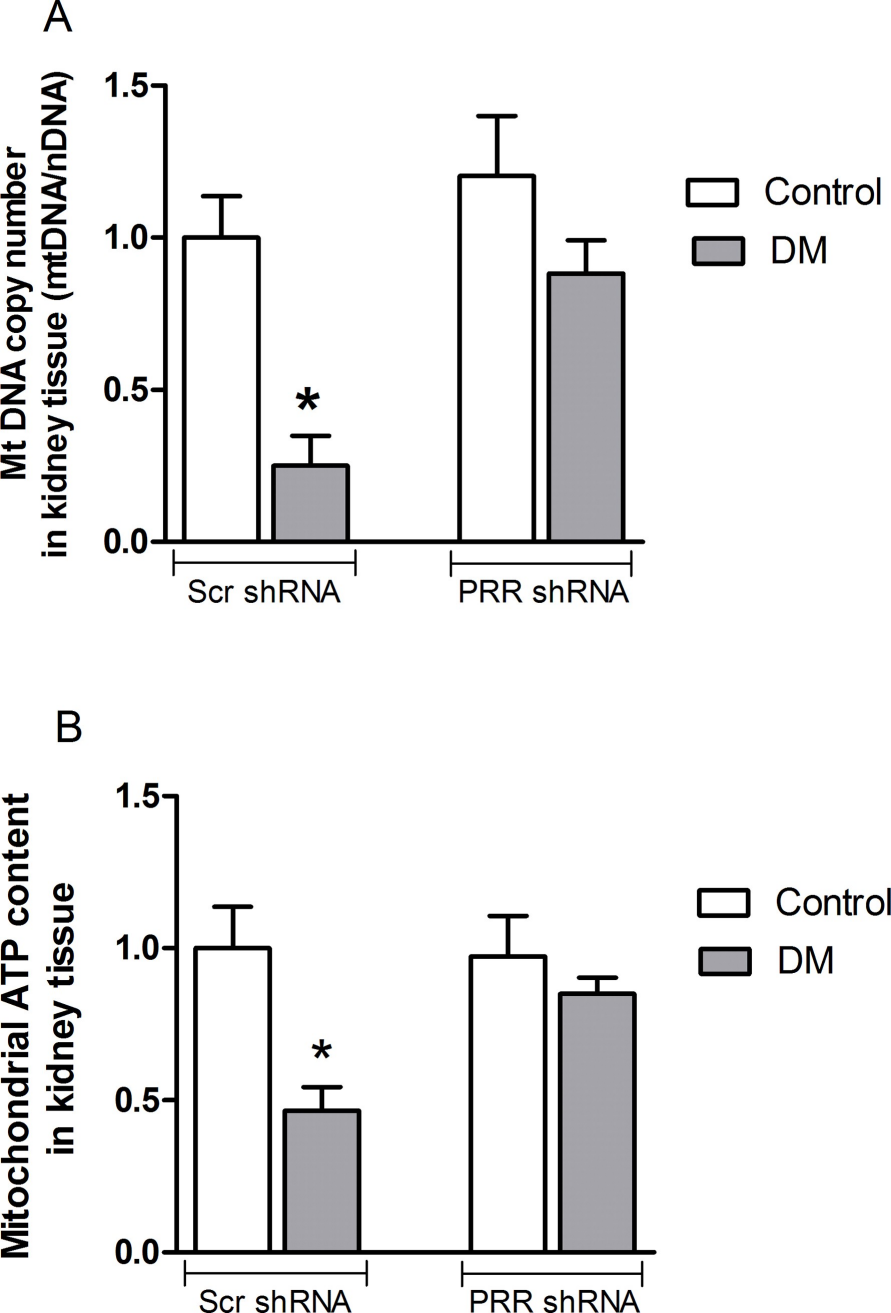
Mitochondrial DNA copy number and ATP level. (A) Mitochondrial DNA copy number and (B) ATP content in renal cortex of normoglycemic control mice treated with scramble (Scr) shRNA (n = 6 each group), and streptozotocin (STZ)-induced diabetic mice (DM; n = 6 each group) treated with PRR shRNA (DM + PRR + shRNA; n = 6 each group), after eight weeks of STZ-induction of diabetes. Data are mean ± SEM. *P < 0.05 vs. Veh + Scr shRNA.

We confirmed mitochondrial biogenesis by quantifying the mitochondrial DNA (mtDNA) copy number. Compared to non-diabetic mice kidney, the DNA copy number was significantly reduced in diabetic mouse kidney by 75% (P<0.05). This reduction of mitochondrial DNA copy number was reversed with PRR shRNA treatment (Fig 5A).

Compared to the non-diabetic kidney, the ATP content in diabetic kidney was significantly decreased by 54% (P<0.05). In contrast, there were no significant differences in ATP level between non-diabetic and diabetic kidney receiving PRR shRNA treatment (Fig 5B).

### Effects of high glucose treatment on the expression of PRR, AMPK, and SIRT-1 in mouse renal mesangial cells

**Fig 6:**
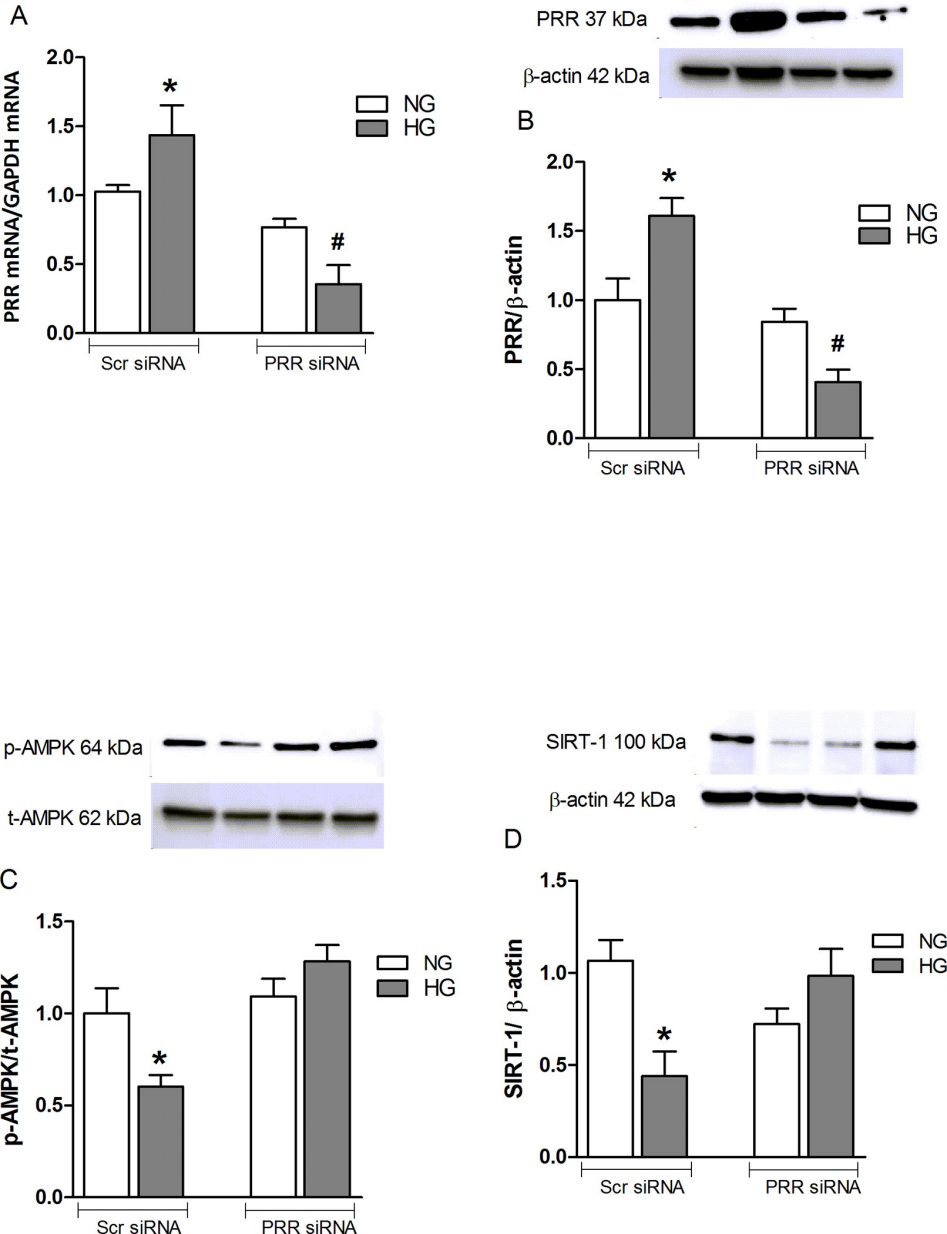
Pro-renin receptor (PRR) expression in mouse renal mesangial cells. (A) Pro-renin receptor (PRR) mRNA and (B) protein expression in normal glucose (NG, 25 mmol L-glucose) and high glucose (HG, 25 mmol D-glucose) cultured mRMCs treated with either scramble (Scr) siRNA or PRR siRNA. Western blot analysis of pAMPK and total AMPK protein expression (C) and SIRT-1 protein expression (D) in normal glucose and high glucose cultured mRMCs treated with either Scr siRNA or PRR siRNA (n = 6 each group). Data are mean ± SEM. *P < 0.05 vs. NG + Scr siRNA; #P < 0.05 vs. HG + Scr siRNA. n = 8 each group.

Compared to normal glucose-treated cells, cells treated with high glucose and scrambled PRR had an increase in PRR mRNA and protein expression by 43 and 61%, (P*<*0.05) respectively. PRR siRNA attenuated high glucose-induced expression of PRR mRNA and protein by 75 and 72%, respectively (P*<*0.05) (Fig 6A and 6B). In contrast, compared with NG + Scr siRNA, HG + Scr siRNA treatment significantly decreased AMPK and SIRT-1 protein expression by 40 and 57% respectively (P< 0.05). PRR siRNA treatment reversed the high glucose-induced reduction in AMPK and SIRT-1 protein expression (Fig 6C and 6D).

### Effects of PRR siRNA or inhibition of AMPK and SIRT-1 individually and combined on the expression of PGC-1α, NRF-1, and mtTFA in mRMCs

**Fig 7:**
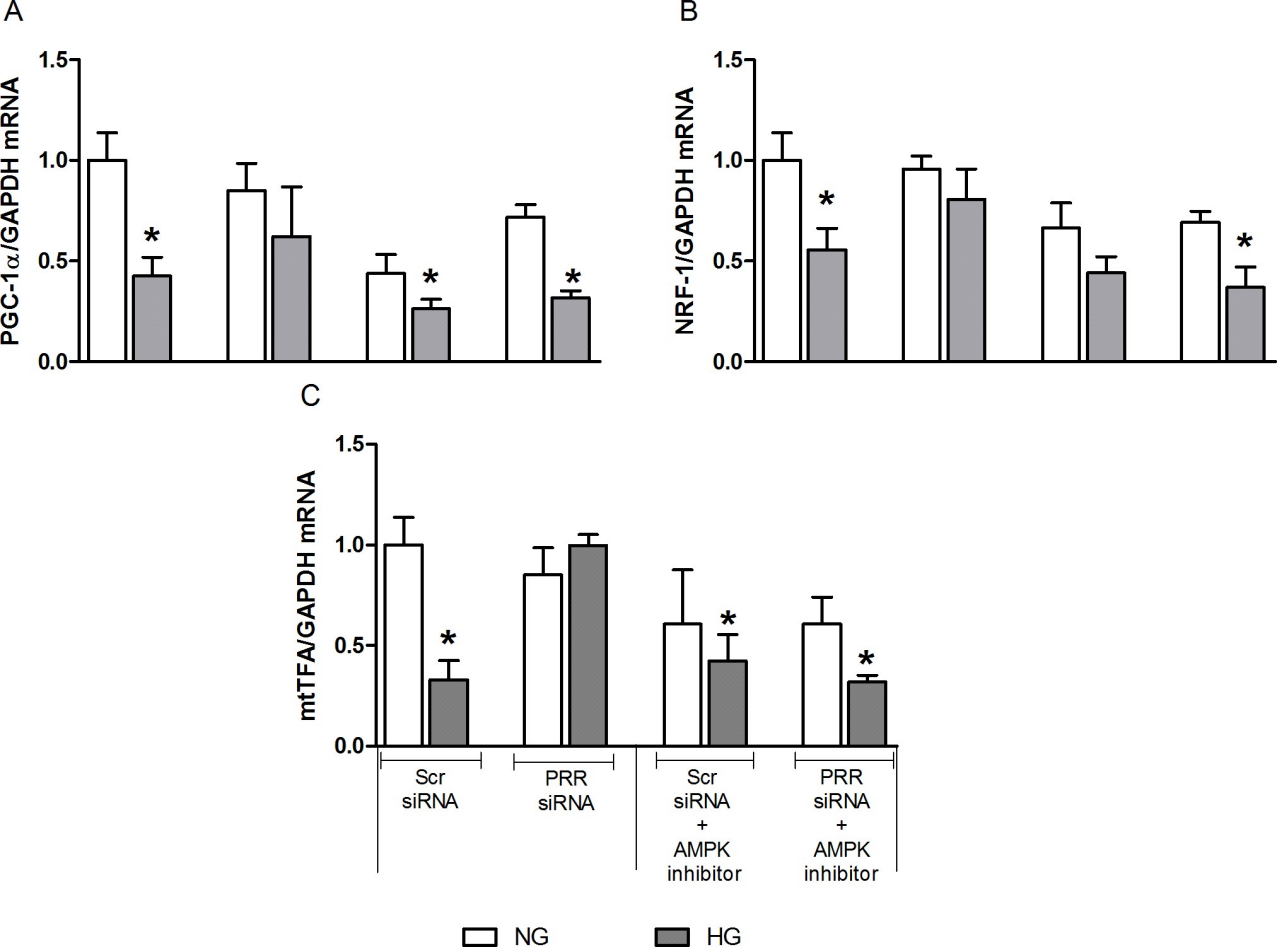
Effects of combined treatment of PRR siRNA and AMPK inhibitor on the expression of PGC-1α, NRF-1, and mtTFA in mRMCs. Effect of AMPK receptor blockade or in combination with (pro)renin receptor (PRR) siRNA on the expression of PGC-1α, NRF-1 and mtTFA mRNA (A-C) in response to NG or HG in mRMCs. Data are mean ± SEM. *P < 0.05 vs. NG + scramble (Scr) siRNA. n = 8 each group.

**Fig 8:**
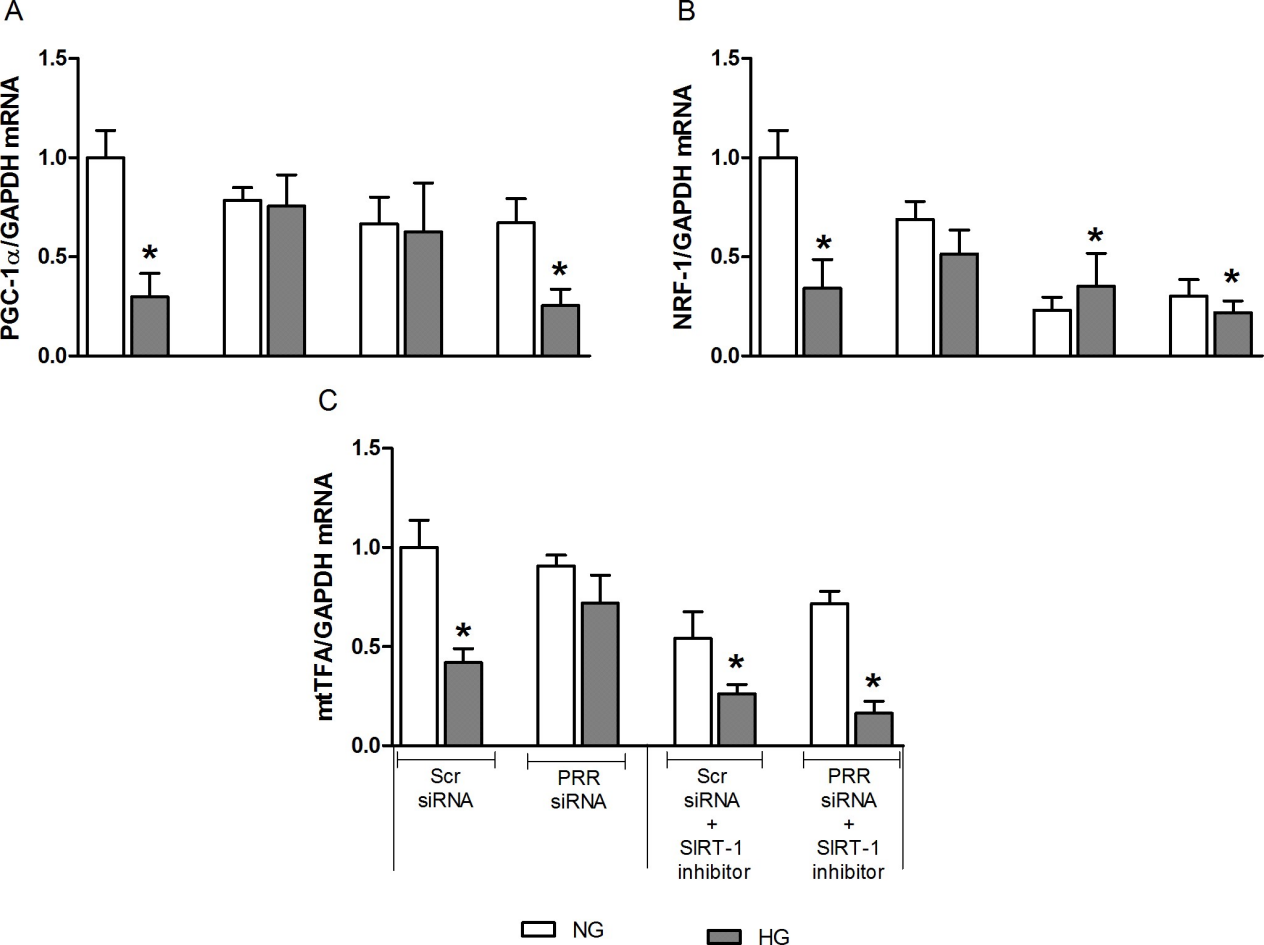
Effects of combined treatment of PRR siRNA and SIRT-1 inhibitor on the expression of PGC-1α, NRF-1, and mtTFA in mRMCs. Effect of SIRT-1 receptor blockade or in combination with (pro)renin receptor (PRR) siRNA, on the expression of PGC-1α, NRF-1 and mtTFA mRNA (A-C) in response to NG or HG in mRMCs. Data are mean ± SEM. *P < 0.05 vs. NG + scramble (Scr) siRNA. n = 8 each group.

Compared to normal glucose, high glucose treatment caused significant decrease in PGC-1α, NRF-1, and mtTFA mRNA expression by 58, 45 and 77%, respectively (all, P*<*0.05). PRR siRNA treatment ameliorated the high glucose-induced decrease in the expression of PGC-1α, NRF-1, and mtTFA mRNA, whereas the HG-induced decrease in PGC-1α, NRF-1, and mtTFA mRNA expression remained unchanged when treated with AMPK or SIRT-1 inhibitor alone. Thereafter, our results demonstrated that PRR regulation of mitochondrial biogenesis in HG condition is mediated by AMPK and SIRT-1 signaling pathway. Compared to NG + Scr siRNA group (Fig 7A–7C), combined treatment of PRR siRNA and AMPK inhibitor, Compound C, resulted in significant reduction of PGC-1α, NRF-1, and mtTFA mRNA expression by 69, 64 and 69%, respectively (all, P *<* 0.05) in HG group. Similarly, compared to NG+ Scr siRNA, treatment with combined PRR siRNA and SIRT-1 inhibitor, EX-527, significantly decreased PGC-1α, NRF-1 and mtTFA mRNA expression by 75, 78 and 84% (P*<* 0.05), respectively (Fig 8A–8C) in HG group.

### Effects of PRR siRNA and AMPK or SIRT-1 inhibition, individually and combined on the mitochondrial DNA copy number in mRMCs

**Fig 9:**
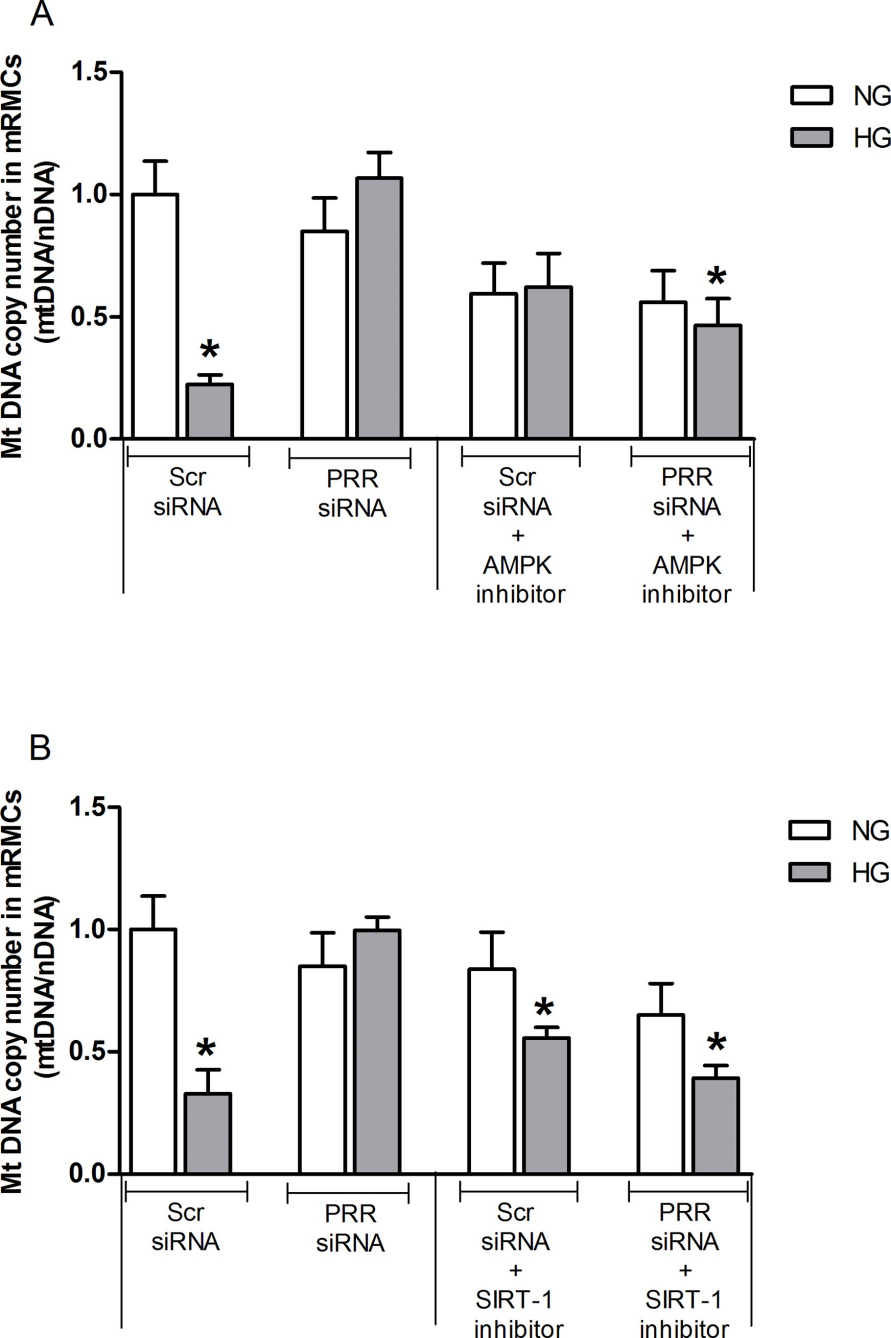
Effects of combined treatment of PRR siRNA and SIRT-1 inhibitor mitochondrial DNA copy number in mRMCs. Effect of (pro)renin receptor (PRR) siRNA alone or in combination with AMPK receptor blockade (A) or SIRT-1 blockade (B) on mitochondrial DNA copy number in response to NG or HG in mRMCs. Data are mean ± SEM. *P < 0.05 vs. NG + scramble (Scr) siRNA. n = 8 each group.

Compared to the NG group, there were significant decreases in mitochondrial DNA copy number by 78% in HG-treated mRMCs (P*<*0.05) which was recovered following PRR siRNA treatment (Fig 9A and 9B). However, mitochondrial DNA copy number remained suppressed in HG treated groups with EX-527 alone. Compared to NG + Scr siRNA, combined treatment of Compound C or EX-527 and PRR siRNA (Fig 9A and 9B) significantly decreased mtDNA copy number by 54 and 61% (both, P<0.05) but the percentage reduction was not different from the levels observed with individual Compound C and EX-527 treatment in HG group.

### Expression of NOX4 in kidney of normoglycemic and STZ-induced diabetic mice and in mRMCs treated with normal and high glucose medium

**Fig 10:**
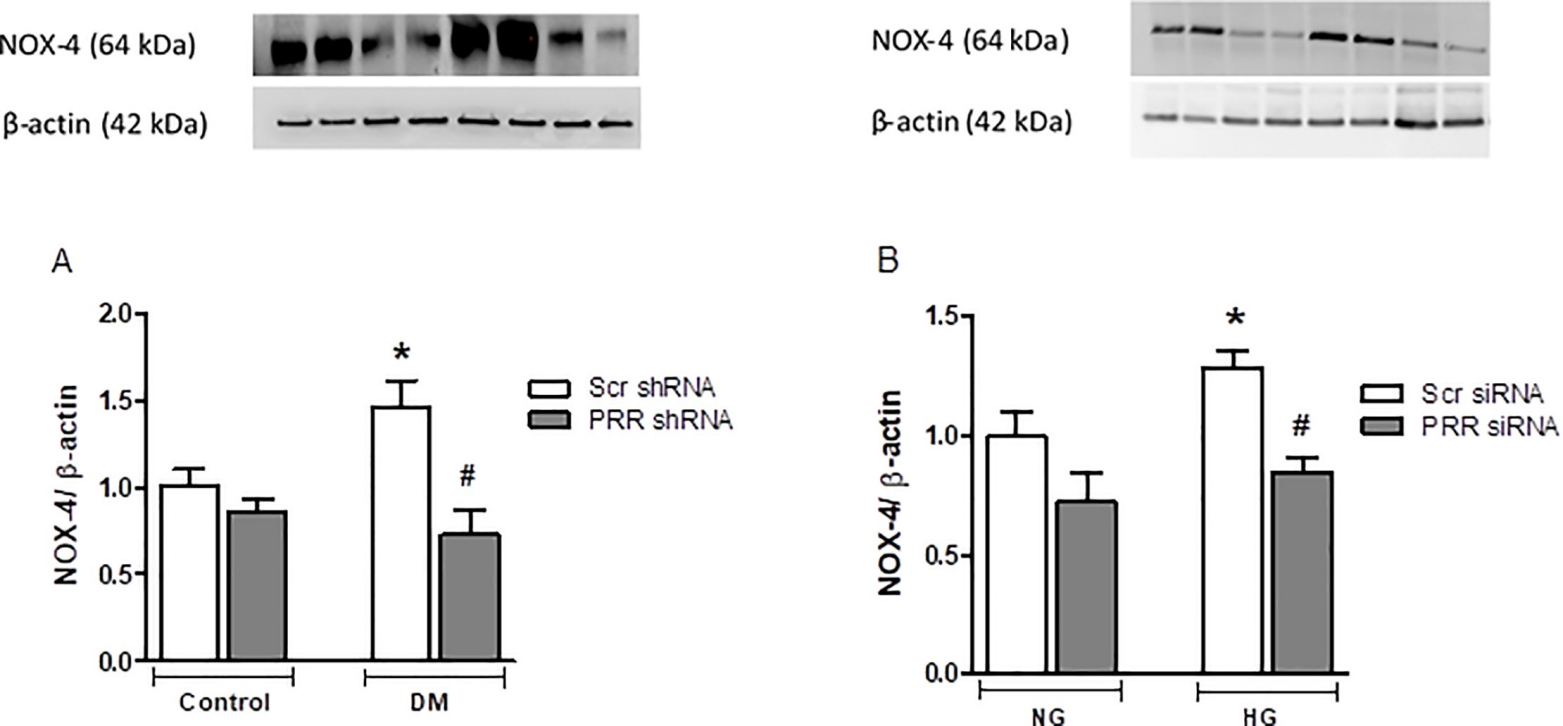
Expression of NOX4 in diabetic mice kidney and in mRMCs. Expression of NOX4 in (A) in renal cortex of normoglycemic control mice treated with scramble (Scr) shRNA (n = 5 each group), and streptozotocin (STZ)-induced diabetic mice (DM; n = 5 each group) treated with PRR shRNA (DM + PRR + shRNA; n = 5 each group), after eight weeks of STZ-induction of diabetes. Data are mean ± SEM. *P < 0.05 vs. Veh + Scr shRNA. #P < 0.05 vs. DM + PRR shRNA, (B) in normal glucose (NG, 25 mmol L-glucose) and high glucose (HG, 25 mmol D-glucose) cultured mRMCs treated with either scramble (Scr) siRNA or PRR siRNA. Data are mean ± SEM. *P < 0.05 vs. NG + Scr siRNA; #P < 0.05 vs. HG + PRR siRNA. n = 6 each group.

We evaluated the mitochondrial NOX-4 protein expression, a marker of oxidative stress, in our diabetic mice and in mRMCs. Compared to non-diabetic mice, NOX-4 protein expression was significantly (P<0.05) higher in diabetic mice by 45%. PRR-KO in diabetic mice significantly (P<0.05) reduced the observed diabetes-induced increase of NOX-4 expression by 49% (Fig 10A). Similarly, our in-vitro experiments demonstrated significant increase in NOX-4 expression by 28% (P<0.05) in cells exposed to high glucose, whereas knockdown of PRR in HG treated cells significantly decreased NOX-4 protein expression (P<0.05), compared to HG treated with Scr siRNA (Fig 10B).

## Discussion

This study was conducted to evaluate the role of PRR on mitochondria biogenesis and function that lead to the development of DKD. We observed that in diabetic mice, renal PRR expression is upregulated and associated with the development of albuminuria, mesangial expansion, and glomerular hypertrophy. These findings are consistent with our previous reports demonstrating increased PRR expression in kidneys of diabetic mouse and in mouse renal mesangial cells in response to hyperglycemia [12, 14]. We also demonstrated a reduction of albuminuria and glomerular hypertrophy in the diabetic mouse with downregulation of PRR expression, confirming the involvement of this receptor in the development of DKD [12]. In the current study, we found reduction in the expression of PGC-1α, NRF-1 and mtTFA, mtDNA copy number and ATP production suggesting decreased mitochondrial biogenesis and function in kidneys of diabetic mice. These findings were reversed with downregulation of renal PRR. These data explain our recent observation that PRR is involved in mitochondrial dysfunction in DKD. Importantly, the current study provided evidence for the involvement of PRR in the reduction of mitochondrial biogenesis via AMPK/SIRT-1/ PGC-1α signaling pathway.

In stressful environment such as hyperglycemia, mitochondria is highly susceptible to damage since mtDNA lacks protection provided by the nucleosomes in the nuclear DNA and does not have a DNA repair mechanism, which can lead to mtDNA depletion [25]. Decreased mitochondrial biogenesis compromises the cells efficient function and thus impacts negatively on health [26, 27]. The present work identified a novel role of PRR in regulating mitochondrial biogenesis and elucidates how this interaction may play an important role in the development of DKD. Our data clearly provided ample evidence in support of this conclusion. PGC-1α, NRF-1 and mtTFA, key regulators stimulating mitochondrial biogenesis, were reduced in the kidneys of STZ-diabetic mouse. Defects in the expression of these mitochondrial biogenesis regulators were found at the mRNA level as well as at the protein level. Subsequently, *in-vitro* experiments also revealed that high-glucose reduced PGC-1α, NRF-1 and mtTFA expression in cultured mRMCs. These results indicate that high levels of glucose decreases mitochondrial biogenesis both *in vivo* and *in vitro*. This is consistent with previous reports showing decreased PGC-1α level in the kidneys of db/db mice [28], and in cultured mesangial cells exposed to 30 mmol/L high glucose [29]. Since mitochondria biogenesis directly controls the mtDNA copy number [30], we demonstrated that diabetic mouse kidney had suppressed renal mtDNA copy number. This suggests that renal mitochondrial biogenesis machinery is impaired in diabetes and thus could have major impact in increasing risk in the development of DKD. In addition, we also observed reduction in ATP production in diabetic kidney, confirming compromised mitochondrial functional in DKD. Since mitochondrial biogenesis and function are typically tightly coupled in the kidney, it is conceivable that mitochondrial damage and decreased mitochondrial density and mtDNA content in DKD could hinder ATP synthesis [31].

Recently, much attention has been focused on developing strategies that can improve mitochondrial function by stimulating the mitochondrial biogenesis pathway and therefore, could provide a promising option for prevention and treatment of DKD. Recently we reported that PRR contributes to the development of diabetic nephropathy and increased mitochondrial oxidative stress [11]. In the current study, we ameliorated the decreased mitochondrial biogenesis in the diabetic kidney by the knockdown of PRR and improved the mitochondrial function. These observations provide strong support showing that PRR plays a crucial role in the regulation and maintenance of mtDNA, and possibly serves as a link between DKD and mitochondrial biogenesis. It also indicates that targeting PRR to improve mitochondrial biogenesis can be a new strategy to prevent DKD.

AMP-activated protein kinase (AMPK) and the Sirtuin-1 (SIRT-1) are the two main energy-sensing molecules that have been reported to directly affect PGC-1α activity through phosphorylation and deacetylation, respectively. Our study demonstrated that downregulation of PRR significantly ameliorated the SIRT-1/AMPK expression in response to high glucose. These data suggest that the effects of PRR downregulation in promoting mitochondrial biogenesis and the amelioration of mitochondrial dysfunction are related to up-regulation of the SIRT-1/AMPK pathway. Combined treatment of PRR siRNA along with AMPK or SIRT-1 blockade caused further reduction in the mitochondrial biogenesis suggesting complementary effects between SIRT-1/AMPK and PRR on mitochondrial biogenesis. Further studies are needed to elucidate the intracellular signaling mechanisms connecting PRR to PGC-1α induced mitochondrial homeostasis. Also, it is certainly possible that PRR may directly regulating AMPK and SIRT-1 in diabetes and in high-glucose induced cells. The exact signal of PRR regulating AMPK is not directly demonstrated in the present study. However, previous studies demonstrated that transforming growth factor-β (TGF-β) and ROS has a direct stimulation of AMPK in kidney disease. In our previous studies, we demonstrated that PRR could be contributing to the reported increase in the renin-angiotensin-system (RAS) activity in diabetes, with subsequent increase in TGFβ-connective tissue growth factor (CTGF) axis [14]. This suggests that PRR may regulate AMPK through activating ROS and TGF-β1 signaling cascade. Nonetheless, the effect of PRR on AMPK and SIRT-1 under HG or diabetes has not been studied and we plan to do this in the future.

In summary, our study elucidates the role of PRR as a new concept linking reduced mitochondrial biogenesis and function to DKD. We demonstrated the involvement of PRR in the defect of AMPK/SIRT-1/PGC-1α signaling cascade, resulting in dysfunctional mitochondrial biogenesis. In conclusion, PRR suppresses mitochondrial biogenesis and function via AMPK/SIRT-1/PGC-1α pathway in DKD.

## Supporting information

S1 Fig Raw western blot image of PRR and β-actin protein expressions in non-diabetic control mice, and streptozotocin (STZ)-induced diabetic mice treated with Scr-and PRR shRNA (correspond to Fig 2B in the manuscript). (PDF)

S2 Fig Raw western blot image of PGC-1α and β-actin protein expressions in non-diabetic control mice, and streptozotocin (STZ)-induced diabetic mice treated with Scr-and PRR shRNA (correspond to Fig 3D in the manuscript). (PDF)

S3 Fig Raw western blot image of NRF-1 and β-actin protein expressions in non-diabetic control mice, and streptozotocin (STZ)-induced diabetic mice treated with Scr-and PRR shRNA (correspond to Fig 3E in the manuscript). (PDF)

S4 Fig Raw western blot image of mtTFA and β-actin protein expressions in non-diabetic control mice, and streptozotocin (STZ)-induced diabetic mice treated with Scr-and PRR shRNA (correspond to Fig 3F in the manuscript). (PDF)

S5 Fig Raw western blot image of p-AMPK and t-AMPK protein expressions in non-diabetic control mice, and streptozotocin (STZ)-induced diabetic mice treated with Scr-and PRR shRNA (correspond to Fig 4A in the manuscript). (PDF)

S6 Fig Raw western blot image of SIRT-1 and β-actin protein expressions in non-diabetic control mice, and streptozotocin (STZ)-induced diabetic mice treated with Scr-and PRR shRNA (correspond to Fig 4B in the manuscript). (PDF)

S7 Fig Raw western blot image of PRR and β-actin protein expressions in response to normal glucose (NG), and high glucose in mRMCs treated with Scr-and PRR siRNA (correspond to Fig 6B in the manuscript). (PDF)

S8 Fig Raw western blot image of p-AMPK and t-AMPK protein expressions in response to normal glucose (NG), and high glucose in mRMCs treated with Scr-and PRR siRNA (correspond to Fig 6C in the manuscript). (PDF)

S9 Fig Raw western blot image of SIRT-1 and β-actin protein expressions in response to normal glucose (NG), and high glucose in mRMCs treated with Scr-and PRR siRNA (correspond to Fig 6D in the manuscript). (PDF)

S10 Fig Raw western blot image of NOX-4 and β-actin protein expressions in non-diabetic control mice, and streptozotocin (STZ)-induced diabetic mice treated with Scr-and PRR shRNA (correspond to Fig 10A in the manuscript). (PDF)

S11 Fig Raw western blot image of NOX-4 and β-actin protein expressions in response to normal glucose (NG), and high glucose (HG) in mRMCs treated with Scr-and PRR siRNA (correspond to Fig 10B in the manuscript). (PDF)
