# Supplementary material for: Pro-renin receptor suppresses mitochondrial biogenesis and function via AMPK/SIRT-1/ PGC-1α pathway in diabetic kidney
Source: PLoS One. 2019 Dec 4;14(12):e0225728. doi: 10.1371/journal.pone.0225728 (PMC6892478; doi:10.1371/journal.pone.0225728)
Supplement: S1 Fig — (PDF) [file pone.0225728.s001.pdf]

Pro-renin receptor suppresses mitochondrial biogenesis and function via AMPK/SIRT-1/ PGC-1α pathway in diabetic kidney

Safia Akhtar and Helmy M Siragy

Department of Medicine, University of Virginia, Charlottesville, Virginia, USA.

Short title: Pro-renin receptor suppresses mitochondrial biogenesis and function

Corresponding author:

Email: hms7a@virginia.edu (HMS)

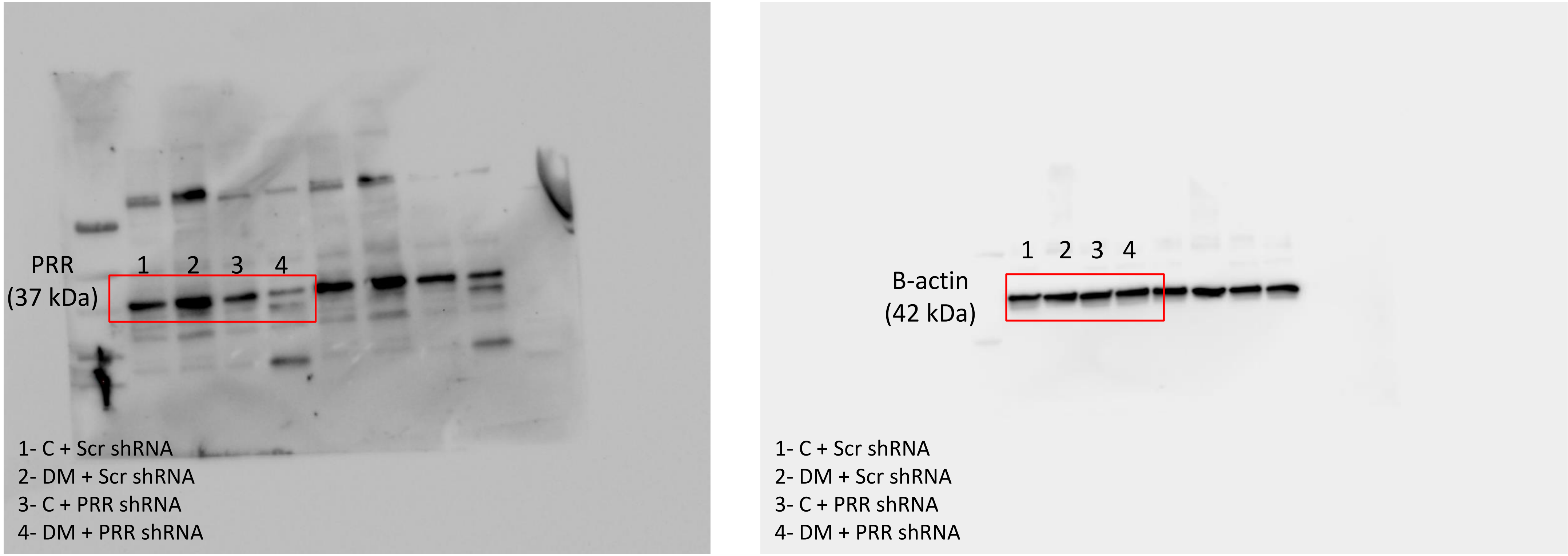

Fig S1: Raw western blot image of PRR and β-actin protein expressions in non-diabetic control mice, and streptozotocin (STZ)-induced diabetic mice treated with Scr-and PRR shRNA (correspond to Fig 2B in the manuscript).
