# Supplementary material for: Pro-renin receptor suppresses mitochondrial biogenesis and function via AMPK/SIRT-1/ PGC-1α pathway in diabetic kidney
Source: PLoS One. 2019 Dec 4;14(12):e0225728. doi: 10.1371/journal.pone.0225728 (PMC6892478; doi:10.1371/journal.pone.0225728)
Supplement: S2 Fig — (PDF) [file pone.0225728.s002.pdf]

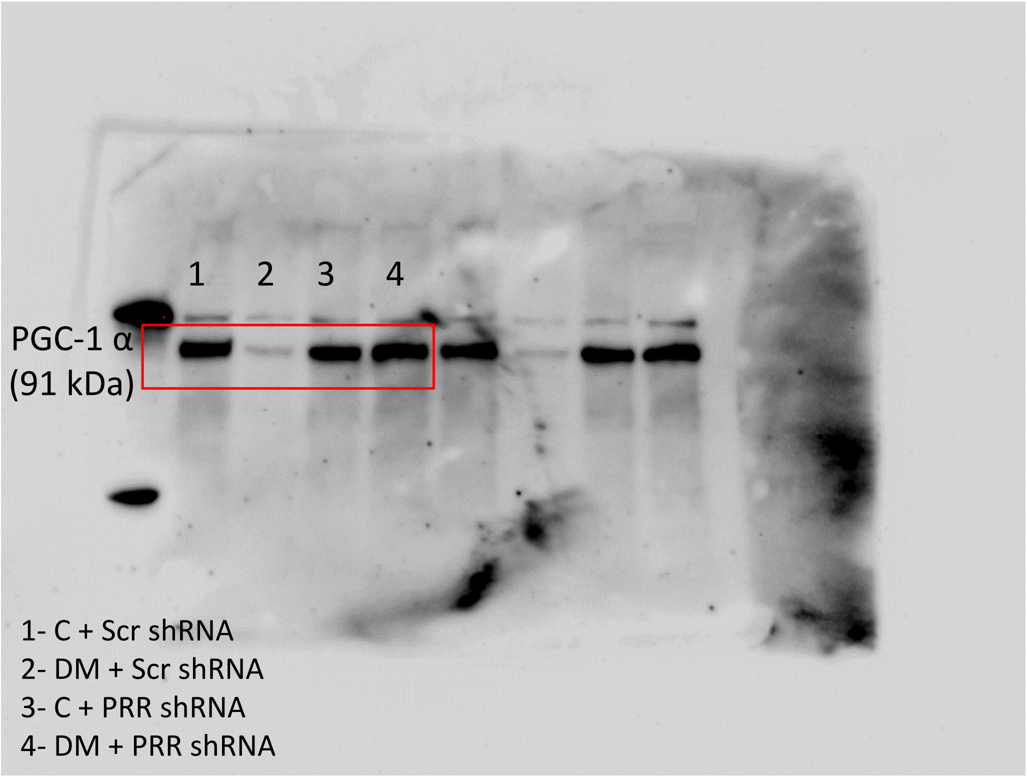

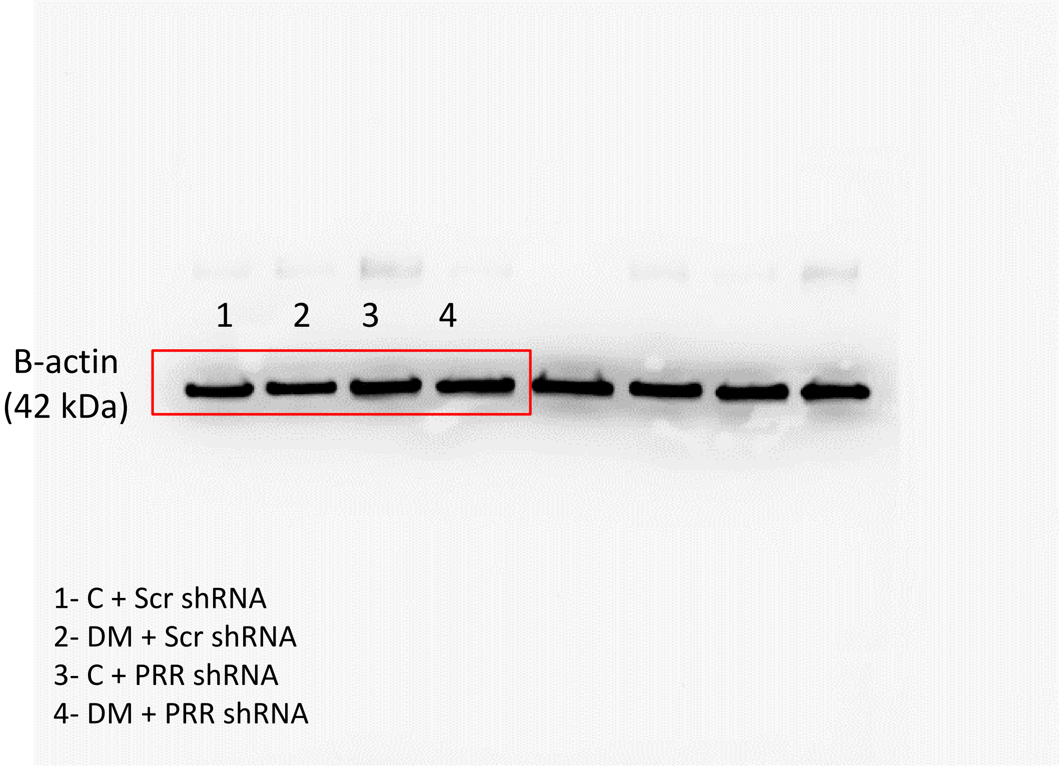

Fig S2: Raw western blot image of PGC-1α and β-actin protein expressions in non-diabetic control mice, and streptozotocin (STZ)-induced diabetic mice treated with Scr-and PRR shRNA (correspond to Fig 3D in the manuscript).
