# Supplementary material for: Pro-renin receptor suppresses mitochondrial biogenesis and function via AMPK/SIRT-1/ PGC-1α pathway in diabetic kidney
Source: PLoS One. 2019 Dec 4;14(12):e0225728. doi: 10.1371/journal.pone.0225728 (PMC6892478; doi:10.1371/journal.pone.0225728)
Supplement: S5 Fig — (PDF) [file pone.0225728.s005.pdf]

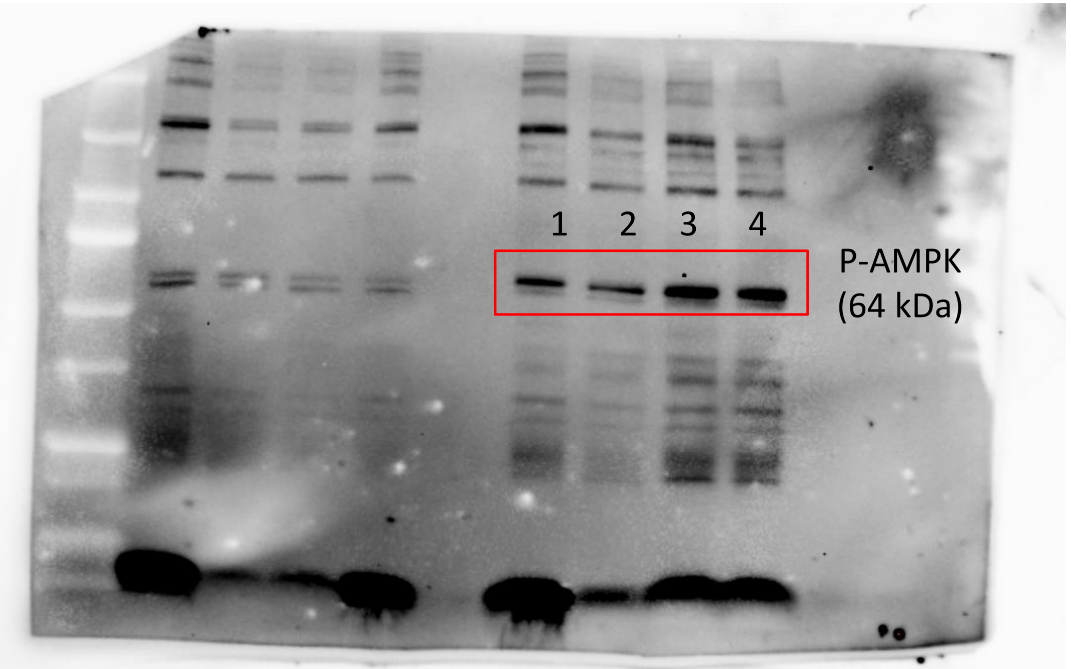

1- C + Scr shRNA
2- DM + Scr shRNA
3- C + PRR shRNA
4- DM + PRR shRNA

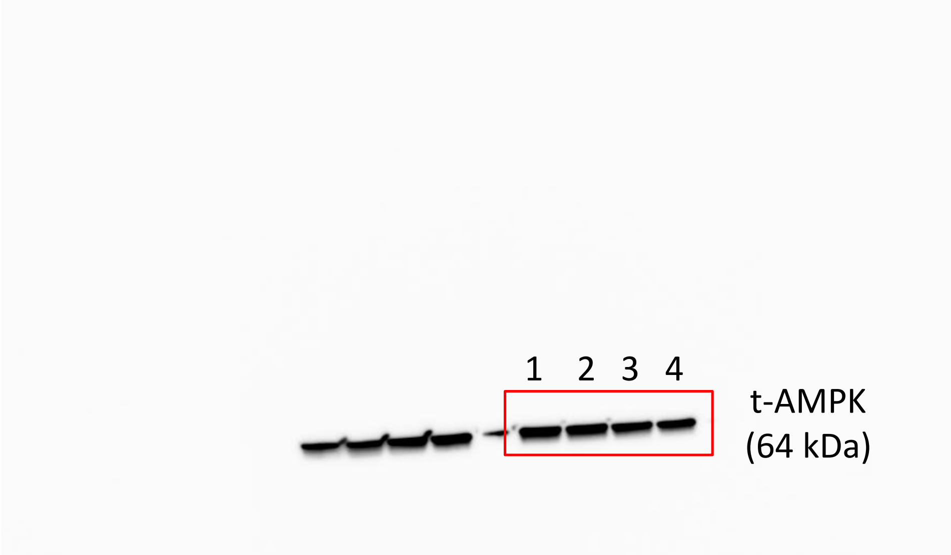

1- C + Scr shRNA
2- DM + Scr shRNA
3- C + PRR shRNA
4- DM + PRR shRNA

Fig S5: Raw western blot image of p-AMPK and t-AMPK protein expressions in non-diabetic control mice, and streptozotocin (STZ)-induced diabetic mice treated with Scr-and PRR shRNA (correspond to Fig 4A in the manuscript).
