# Supplementary material for: Pro-renin receptor suppresses mitochondrial biogenesis and function via AMPK/SIRT-1/ PGC-1α pathway in diabetic kidney
Source: PLoS One. 2019 Dec 4;14(12):e0225728. doi: 10.1371/journal.pone.0225728 (PMC6892478; doi:10.1371/journal.pone.0225728)
Supplement: S7 Fig — (PDF) [file pone.0225728.s007.pdf]

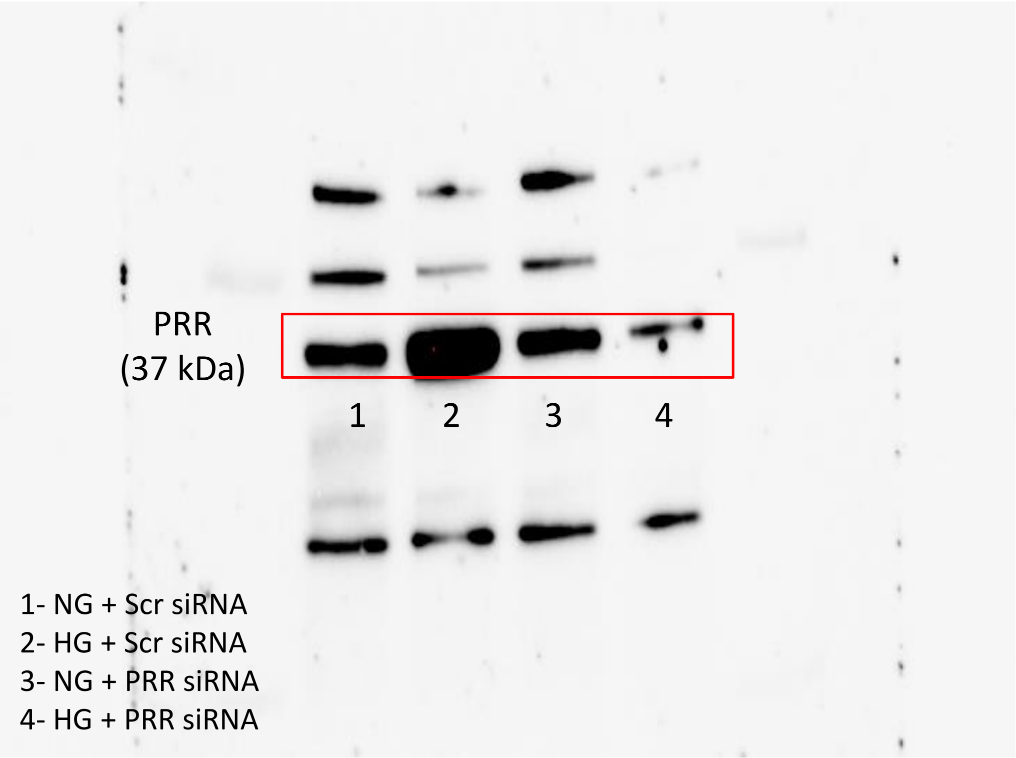

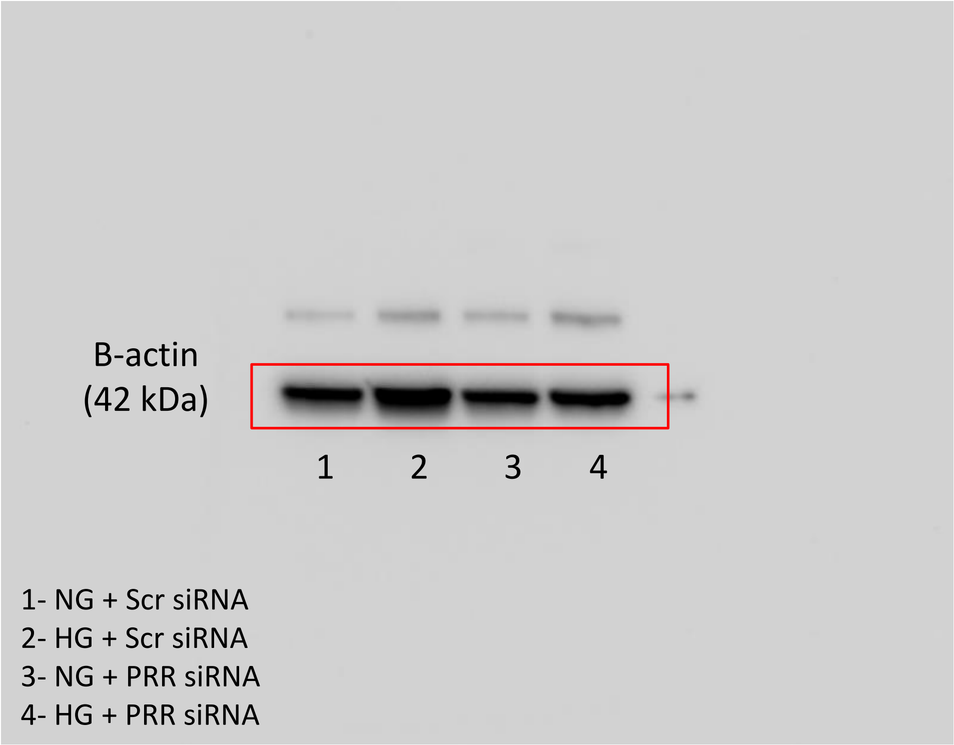

Fig S7: Raw western blot image of PRR and β-actin protein expressions in response to normal glucose (NG), and high glucose (HG) exposed mRMCs treated with Scr-and PRR siRNA (correspond to Fig 6B in the manuscript).
